# Effect of Polyvalence on the Antibacterial Activity of a Synthetic Peptide Derived from Bovine Lactoferricin against Healthcare-Associated Infectious Pathogens

**DOI:** 10.1155/2018/5252891

**Published:** 2018-06-10

**Authors:** Sandra C. Vega Chaparro, J. Tatiana Valencia Salguero, Diana A. Martínez Baquero, Jaiver E. Rosas Pérez

**Affiliations:** ^1^Pharmacy Department, Science Faculty, Universidad Nacional de Colombia, Carrera 30 No. 45-03, Zip Code 11321, Bogotá, Colombia; ^2^Bacteriology Department, Science of Health Faculty, Universidad Colegio Mayor de Cundinamarca, Calle 28 No. 5B-02, Zip Code 110311, Bogotá, Colombia

## Abstract

Antimicrobial peptides (AMPs) are gaining interest as potential therapeutic agents. Peptides derived from bovine lactoferricin B (LfcinB) have been reported to exhibit antimicrobial activity, and the LfcinB RRWQWR sequence is the smallest known motif that exhibits antibacterial and cytotoxic activity. Our goal was to examine the effect of multicopy arrangements of the RRWQWR motif, on its antibacterial activity against healthcare-associated infections (HCAIs). Linear and branched peptides containing the RRWQWR motif were generated using solid phase peptide synthesis-Fmoc/tBu methodology, purified, and characterized using reverse phase-high performance liquid chromatography and matrix-assisted laser desorption/ionization time of flight mass spectrometry. For each peptide, the antibacterial activity against* Staphylococcus aureus* (ATCC 25923 and 33591 strains) and* Klebsiella pneumoniae* (ATCC 13883 and 700603 strains) was assessed by measuring the minimum inhibitory and the minimum bactericidal concentrations, in the exponential phase. Cells were observed by scanning electron microscopy, and the hemolytic activity of the peptides was assessed. The overall results demonstrate that, compared to linear analogues, polyvalent presentation of the RRWQWR motif enhances its antibacterial activity against both Gram-negative and Gram-positive bacteria even on resistant strain.

## 1. Introduction

Until very recently, “nosocomial” was the term used to refer to any disease acquired by a patient under medical care [1], particularly with reference to infections acquired by hospitalized patients. Recently, a new expression, “healthcare-associated infections” (HCAIs), has been established to refer to infections due to hospitalization. HCAIs are a major risk factor for serious health problems and can lead to death [2].

Several microbes, such as protozoans, fungi, viruses, and mycobacteria, can cause HCAIs, but bacteria are responsible for approximately 90% of the infections [3]. The incidence of various bacteria in HCAI infection changes over time and depends on the region [1]. Currently, pathogens that commonly caused HCAIs include* Staphylococcus aureus*,* Klebsiella pneumoniae, Pseudomonas aeruginosa, Escherichia coli*, and* Enterococcus faecalis *[4].

Furthermore, the excessive and improper use of traditional broad-spectrum antibiotics, especially in healthcare venues, increases the prevalence of bacteria resistant to conventional antibiotics and has resulted in an increase in HCAI infections caused by drug-resistant pathogens [1]. The design of new antimicrobial molecules effective against resistant bacteria is crucial to overcome and control the antibiotic resistance.

One promising group of potential lead structures for antibiotics is antimicrobial peptides (AMPs), as they are naturally derived compounds with antimicrobial activity. The rational design of new AMPs offers hope for enhanced biological activity and cheaper, more-efficient production. Rational design methodologies include in silico methodologies. Large-scale, high-quality recombinant production can be done using tobacco mosaic virus and gene-editing techniques such as CRISPR (Clustered Regularly Interspaced Short Palindromic Repeats) recombinant peptide biosynthesis [5]

In this AMP group, peptides derived from LfcinB protein have been reported to exhibit antimicrobial activity. Peptides derived from bovine lactoferricin B (LfcinB) have been reported to exhibit antimicrobial activity [6]. The RRWQWR sequence of LfcinB is the smallest motif that exhibits antibacterial activity [7, 8], as well as cytotoxic effect [9]. Previous studies of our group have shown that this sequence motif has antibacterial activity although it is minimal [6, 8, 9]. Our goal was to examine the effects of various arrangements of multiple copies of the RRWQWR motif on its antibacterial activity against HCAI bacteria.

## 2. Materials and Methods

### 2.1. Microorganisms

All microorganisms were purchased from the American Type Culture Collection (ATCC). To determine the antibacterial activity of synthetic peptides against HCAI bacteria, the* Staphylococcus aureus *strains ATCC 25923 and ATCC 33591 were employed as sensitive and resistant strains, respectively, of a Gram-positive microorganisms species, and* Klebsiella pneumoniae* ATCC 13883 and ATCC 700603 as sensitive and resistant strains of a Gram-negative species.

### 2.2. Antibacterial Peptides

**Figure 1:**
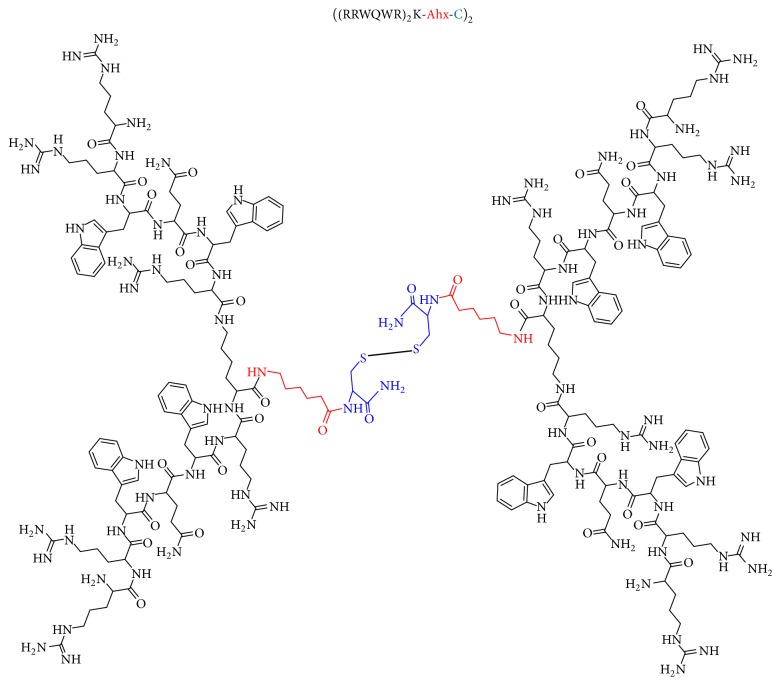
Representation of the tetrameric RRWQWR motif peptide molecule.

To improve the antibacterial activity of the RRWQWR motif against* S. aureus* and* K. pneumoniae*, our approach was based on presenting motif repetitions as linear or branched derived peptides [10]. The peptides were synthesized using solid phase peptide synthesis- (SPPS-) Fmoc/tBu methodology, as previously reported by our group [8, 9]. Bearing in mind that the amidated peptides have greater biological activity, the peptides synthetized and evaluated in this work were carried out using Rink-amide as a solid support, in order to obtain peptides with amide function at their carboxyl end. The purity of the peptides was >90%, as determined via reverse phase-high performance liquid chromatography (RP-HPLC) analysis. All peptides had the expected molecular weight, as verified by matrix-assisted laser desorption/ionization time of flight mass spectrometry (MS MALDI-TOF). Synthetized peptides were stored as lyophilized products. To obtain the dimeric peptide, di-FMOC-protected lysine was used, which enabled simultaneous synthesis of the two peptide chains (one from the *α*-amino group and the other, from the *ε*-amino group of this amino acid) [6]. The tetrameric peptide was obtained through disulfide bond formation (Figure 1). Briefly, the purified peptide (RRWQWR)_2_-K-Ahx-C was oxidized using 10% dimethyl sulfoxide in a buffer PBS at pH 7.5 at room temperature, in accordance with Leon-Calvijo et al. [6].

The peptides molecules were synthesized and characterized by the “Síntesis y Aplicación de Moléculas Peptídicas (SAMP)” research group of the Faculty of Science of the Universidad Nacional de Colombia.

### 2.3. Determination of Minimum Inhibitory Concentration (MIC) and Minimum Bactericidal Concentration (MBC)

The antibacterial activity assay for peptides and controls was performed according to the National Committee for Clinical Laboratory Standards method M7-A7 [11]. MIC and MBC values were determined using a broth microdilution and growth inhibition method [12] with some modification. Briefly, MIC assays were performed by liquid inhibition growth assay in an untreated sterile 96-well flat-bottom tissue culture plate. Bacteria were cultured overnight on Mueller-Hinton agar (MHA). Three colonies were transferred to 8 mL of Mueller-Hinton broth (MHB) and incubated at 37°C until the mid-exponential phase of growth. The turbidity of the cultures was measured and adjusted spectrophotometrically to McFarland standard 0.5, then diluted to a final concentration of 5 × 10^7^ colony forming units (CFU)/well. Synthetic antibacterial peptide candidate stock solutions were diluted to final concentrations per well of 200, 100, 50, 25, 12.5, and 6.25 *μ*M. Each concentration was evaluated in duplicate in three repetitions of the assay.

Wells containing MHB with bacterial inoculum only served as bacterial growth controls. Additional controls included MHB alone and MHB with ciprofloxacin (CIP) (2 *μ*g/mL) and bacteria as a positive control. The microplate was incubated for 24 h at 37°C, and growth inhibition was examined by monitoring the optical density at 620 nm (OD_620_). MIC was defined as the lowest peptide concentration that inhibited bacteria growth >90%.

To determine the MBC, an aliquot from each well of the MIC assay was spread onto MHA. After 18 h at 37°C, the concentration that inhibited bacterial growth was determined. Each of these tests was performed four times. MBC was defined as the lowest concentration of peptide that reduced the number of bacteria by 99.9%* in vitro *[12].

### 2.4. Electron Microscopy

Scanning electron microscopy (SEM) was used to observe bacterial morphology.* S. aureus *and* K. pneumoniae *strains were grown to mid-logarithmic phase and adjusted spectrophotometrically to the McFarland 0.5 standard, corresponding to ~1 × 10^8^ CFU/mL. Subsequently, 1 mL of bacterial suspension was distributed into 3 tubes, and peptides (dimeric and tetrameric molecules) were added to two tubes at 3x the MIC. The third culture, without peptide, was used as a control. Samples were incubated aerobically at 37°C for 2 h, and the bacterial suspension was centrifuged at 1459 ×g for 3 min and washed twice with Millonig's Phosphate Buffer (0.10 M, pH 7.4). For SEM, each sample was fixed with 1 mL of 2.5% glutaraldehyde at 4°C for 2 h. The fixed samples were dehydrated in an ethanol gradient (50, 70, 80, 90, and 100%) for 20 min and centrifuged at 1459 ×g for 10 min. The bacterial pellet was suspended in 100% ethyl alcohol and air-dried. Finally, the slides were taped onto stubs, coated with gold using a Quorum Q150R sputter coater, and observed with an FEI Quanta 200-r SEM.

### 2.5. Haemolytic Activity

Human erythrocytes collected from the blood samples of healthy humans were harvested by centrifugation for 7 min at 162 ×g and washed three times in phosphate-buffered saline (PBS). The erythrocytes (2% haematocrit in PBS) were incubated with peptide molecules at several concentrations (6.25, 12.5, 25, 50, and 100 *μ*M), for 2 h at 37°C. PBS was used as a negative control, and sterile distilled water was used as a positive control for haemolysis (100% of haemolysis). The plate was subsequently centrifuged at 1459 ×g for 10 min at 4°C. Aliquots of the supernatants of each well (75 *μ*L) were carefully transferred to a new sterile 96-well plate, and haemolytic activity was evaluated by measuring the OD_492_ using an Asys Expert Plus Microplate reader. The experiments were performed in duplicate, and peptide haemolysis activity was calculated.

## 3. Statistical Analysis

Data were analyzed using SPSS 11.0 software and are presented as the mean ± standard deviation.

## 4. Results

### 4.1. Antibacterial Peptides

For this research, five peptides (Table 1) containing the RRWQWR motif were synthesized through SPPS, using the manual Fmoc/tBu strategy. Specifically, we generated a linear LfcinB motif (20–25), a palindromic sequence (PLS), and LfcinB (17–31), which was considered an antibacterial peptide reference, according to results previously reported by Leon-Calvijo et al. [6]. Palindromic sequence is not really palindromic as it is but it shifted sequence in order to increase the size of the motif peptide in a linear designed while maintaining the same net charge. Additionally a dimeric peptide was synthesized and a tetrameric peptide as branched derived. Peptide solutions were prepared in sterile water for injection, sterilized by 0.22 *μ*m filtration, and stored at −20°C.

### 4.2. Antibacterial Activity of the Designed Peptide Molecules

We examined the antibacterial activity of the peptides at several concentrations. Specific dose-response profiles were observed for each peptide molecule, and the profiles differed according to the strain evaluated and its sensitivity to antibiotics.

### 4.3. Determination of MIC and MBC

**Figure 2:**
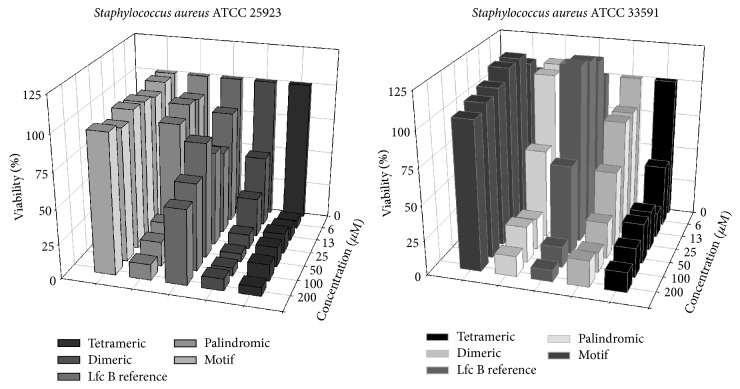
Dose-dependent effects of the peptides on two* Staphylococcus aureus* strains.

Figure 2 shows the specific antibacterial activity profiles for each peptide against representative sensitive and resistant strain of the Gram-positive microorganism* S. aureus*. The peptide motif had no significant effect on either strain. However, the palindromic peptide in general displayed antibacterial activity at concentration ≥74 to 148 *μ*g/mL (≥50 to 100 *μ*M) on both the sensitive and resistant strains. Similarly, the LfcinB reference peptide, which was previously reported to exhibit antibacterial activity on other bacterial pathogens, displayed antibacterial activity, but only affected the resistant strain, at concentration ≥ 100 *μ*g/mL (≥50 *μ*M). Lower, concentrations of this molecule did not exhibit antibacterial activity, and only concentrations ≥ 199 *μ*g/mL (≥100 *μ*M) exhibited significant antibacterial effects. The dimeric and tetrameric peptide molecules exhibited a strong effect on bacterial growth, with dose-dependent effects on both the sensitive and resistant* S. aureus, *and the strongest effect was observed on the sensitive strain, at concentration ≥ 14 *μ*g/mL (≥6.25 *μ*M) and ≥29 *μ*g/mL (≥12.5 *μ*M), respectively.

Concentration expressed in *μ*g/mL for each peptide is reported in Table 2.

**Figure 3:**
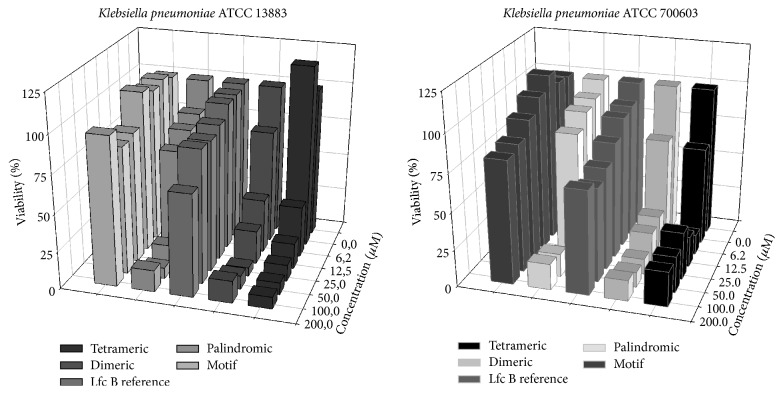
Dose-dependent effects of the peptides on two* Klebsiella pneumoniae* strains.

Conversely, results obtained with Gram-negative* K. pneumonia* were similar for both strains tested (sensitive and resistant) with all analyzed peptide molecules.  Figure 3 shows the specific antibacterial activity profile of each peptide against* K. pneumoniae*. The peptide motif had no substantial effect on either strain, at any concentration. The palindromic peptide exhibited similar antibacterial activity against the sensitive strain at concentration ≥74 *μ*g/mL (≥50 *μ*M); however the resistant strain required a higher concentration to induce similar antibacterial activity ≥148 *μ*g/mL (≥100 *μ*M). The LfcinB reference peptide exhibited similar antibacterial activity to the motif peptide on both strains, while the branched dimeric and tetrameric peptides both exhibited strong antibacterial activity at concentrations ≤27 *μ*g/mL (≤12,5 *μ*M) and ≤57 *μ*g/mL (≤12,5 *μ*M), respectively, against both sensitive and resistant* K. pneumoniae*.

Concentration expressed in *μ*g/mL for each peptide is reported in Table 2.

According to these results, molecules containing repetitions of the RRWQWR motif exhibited antibacterial activity against both sensitive and resistant strains of Gram-positive and Gram-negative bacteria, in the following order:

(1) $$\begin{array}{c} Tetrameric>Dimeric>Palindromic \end{array}$$

The MIC for each peptide against sensitive and resistant strains of* S. aureus* and* K. pneumoniae* were measured using a broth microdilution assay. The MIC value for the five peptide molecules studied against the infecting organisms is shown in Table 2.

The MIC values for the RRWQWR motif showed no antibacterial activity against* S. aureus* and* K. pneumoniae* (>197 *μ*g/mL–200 *μ*M) (Figures 2 and 3; Table 2). Similar results were observed for the LfcinB reference peptide (>398 *μ*g/mL–200 *μ*M), except for the* S. aureus*-resistant strain, against which significant antibacterial activity was demonstrated (≥100 *μ*g/mL–≥50 *μ*M, Table 2). The palindromic peptide had a higher MIC for the sensitive strain (≥74 *μ*g/mL–≥50 *μ*M, Table 2), than the resistant strain (≥9 *μ*g/mL–≥6,25 *μ*M, Table 2), and was more effective against the resistance strain. Remarkably, the dimeric and tetrameric peptides exhibited the strongest inhibitory activities against both* S. aureus* and* K. pneumoniae*.

For* S. aureus*, the dimeric (≥14 *μ*g/mL–≥6.25) and tetrameric (≥29 *μ*g/mL–≥6.25 *μ*M) peptides exhibited greater antibacterial activity than the palindromic on the sensitive strain (Table 2). By contrast, the palindromic (MIC ≥ 9 *μ*g/mL–≥6.25 *μ*M) peptide showed increased activity compared to the tetrameric (MIC ≥ 57 *μ*g/mL–≥12.5 *μ*M) and the dimeric peptide (MIC ≥ 55 *μ*g/mL–≥25 *μ*M), against the resistant strain. Interestingly, the palindromic peptide displayed a difference in the MBC obtained with the sensitive (≥148 *μ*g/mL–≥100 *μ*M) and resistant (≥74 *μ*g/mL–≥50 *μ*M) strains, requiring higher dose to induce the same effect, whereas the branched molecules dimeric (≥110 *μ*g/mL–≥50 *μ*M) and tetrameric (≥57 *μ*g/mL–≥12.5 *μ*M) induced stronger antibacterial effects on both sensitive and resistant strains (Table 2).

For* K. pneumoniae*, the palindromic (≥74 *μ*g/mL–≥50 *μ*M) and dimeric (≥27 *μ*g/mL–≥12,5 *μ*M) peptides did not exhibit any differences in MIC on the sensitive and resistant strains, while tetrameric peptide induced higher antibacterial effects on the sensitive (≥29 *μ*g/mL–≥6,25 *μ*M) that resistance strain (≥57 *μ*g/mL–≥12,5 *μ*M).

The MBC values for each peptide were described as a function of the MBC of the tetrameric peptide, because this molecule exhibited the greatest antibacterial activity on the strains tested in this study.

For* S. aureus*, the tetrameric MBC was ≥57 *μ*g/mL (≥12.5 *μ*M). Therefore, the efficacy profile expressed in concentration unit (*μ*g/mL) for this organism was tetramer (1x MBC) > dimer (1.9x MBC) ≥ palindromic (2.6x MBC and 1.3x MBC for the sensitive and resistant strain, respectively) with similar effects on the sensitive and resistant strains, with the exception of the palindromic molecule.

For* K. pneumonia, *the tetrameric MBC was ≥115 *μ*g/mL (≥25 *μ*M). Therefore, the efficacy profile expressed in concentration unit (*μ*g/mL) for this organism was tetramer (1x MBC) ≥ dimer (0.5x MBC and 1.9x MBC for the sensitive and resistant strain, respectively) > palindromic (1.3x MBC), with similar effects on the sensitive and resistant strains, with the exception of the dimeric molecule.

In the case of* K. pneumoniae*, the palindromic, dimeric, and tetrameric peptides did not exhibit dramatic differences in the MBCs obtained with the sensitive and resistant strains, and the branched molecules (dimeric and tetrameric) induced stronger antibacterial effects.

### 4.4. SEM

**Figure 4:**
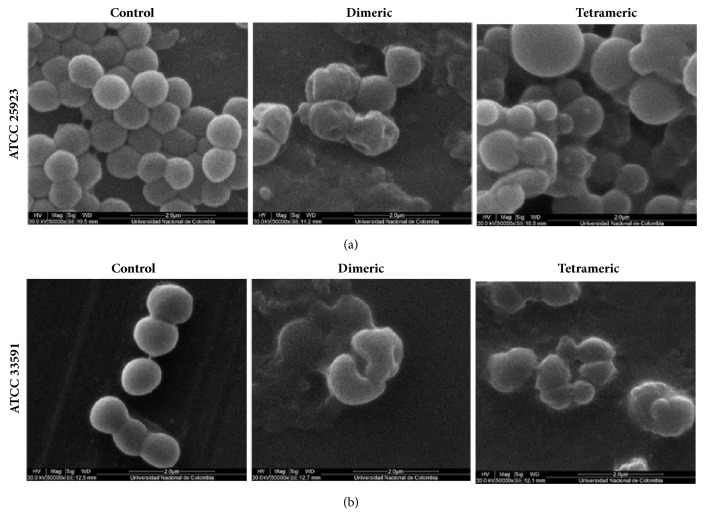
Scanning electron microscopy (SEM) images of* Staphylococcus aureus* after treatment with dimeric and tetrameric peptides.

To examine how the dimeric and tetrameric peptides affect* S. aureus* and* K. pneumoniae*, the morphologies of the microorganisms were observed using SEM analysis. As shown in Figure 4, untreated (control)* S. aureus* was spherical, with a smooth surface and minimal mucus and aggregated in grape-like clusters in both the sensitive and resistant strains. Sensitive* S. aureus *(Figure 4(a)), after treatment with dimeric peptide for 2 h, organized into short chains of cells with morphological alterations such as pitted and wrinkled surfaces. Tetrameric peptide treatment of the same strain induced a reduced number of cells, heterogeneous appearance, and alterations of the bacterial surface. The tetramer induced aggregates of several spherical sizes, and protrusions formed on the bacterial surface. The resistant* S. aureus* strain (Figure 4(b)) exhibited a reduction in the bacterial cell population with both treatments. Dimeric peptide treatment induced cell membrane alterations on the surface and abnormal cell shapes. The tetrameric peptide resulted in abnormal cell shapes and aggregations, along with surface changes. With both treatments, the leakage of cellular contents may have contributed to the creation of the observed aggregates.

**Figure 5:**
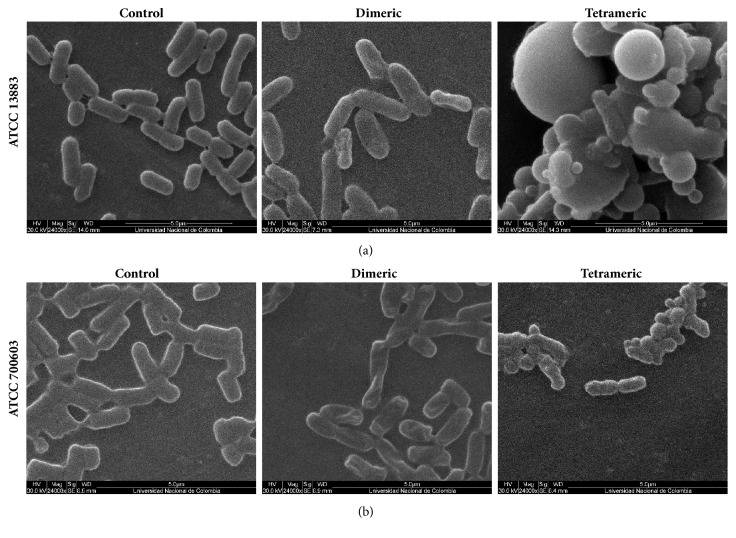
Scanning electron microscopy (SEM) images of* Klebsiella pneumoniae* after treatment with dimeric and tetrameric peptides.

The untreated (control) sensitive strain of* K. pneumoniae* resembled typical coccobacilli, with an encapsulated, smooth surface, and a population of heterogeneous size and shape (Figure 5). After 2 h treatment with dimeric peptide, sensitive* K. pneumonia *(Figure 5(a)) exhibited bacilli with less-defined capsules and morphological alterations such as loss of shape and ruptured cell membranes, generating bacilli with irregular edges. Treatment with the tetrameric peptide induced a reduction in cell number, a very heterogeneous population, and formation of protusions on the bacterial surfaces. The tetramer induced total loss of typical bacilli shape and the formation of aggregates of several shapes and sizes. The untreated resistant* K. pneumoniae *strain (Figure 5(b)) exhibited a bright capsule, mucus, and a smooth surface with no visible damage, whereas dimeric peptide treatment induced cell membrane alterations on the surface such as shrivelling and reduced brightness, as well as decreased size and bacterial population. The effects of tetrameric peptide were even more dramatic, with surface changes and the aggregation of cells of different shapes. In both treatments, the leakage of cellular contents may have contributed to the creation of the observed aggregates.

### 4.5. Haemolytic Activity

**Figure 6:**
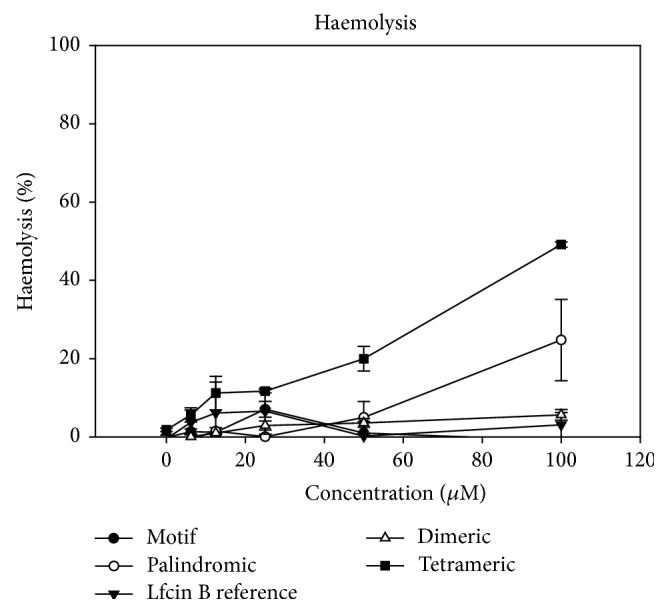
*Haemolytic activity of antibacterial synthetic peptides derived from LfcinB*. Symbols indicate mean and range (*n* = 2).

Lastly, to evaluate the effects of the peptides on normal human erythrocytes, their haemolysis activity was investigated (Figure 6). The percentage of haemolytic activity on human red blood cells after 2 hours was determined via the standard microtitre dilution method. The peptide concentration is reported as *μ*M.

Figure 6 demonstrates that none of the peptides studied reached HC_50_ (the concentration that induces the lysis of 50% of human erythrocytes). The palindromic and the tetrameric peptides exhibited greater haemolysis activity (24.8% and 49.1%, respectively) at the highest peptide concentration tested (100 *μ*M) by inducing permeabilization of the human erythrocytes. Looking at the results in detail (Figure 6), the RRWQWR motif showed no haemolysis activity, and the maximum value (7.1%) of haemolysis activity was obtained with 25 *μ*M peptide. Similarly, the LfcinB reference peptide exhibited the greatest haemolysis value (7,9%) at 12.5 *μ*M. Interestingly, the dimeric peptide exhibited the lowest haemolytic profile at all concentrations tested. In summary, linear and tetrameric motif increased the haemolytic activity; however, the dimeric motif exhibited low haemolysis activity (<5%). Thus the haemolytic activity profiles for the peptides tested here were tetrameric > palindromic > dimeric > LfcinB reference > motif.

## 5. Discussion

AMPs are fascinating prospect for novel antibiotics because of their broad-spectrum activity, including against drug-resistant bacteria LfcinB-derived peptides which are an excellent example. Previous work has shown that how structural changes to the RRWQWR motif can influence the antimicrobial activity of the resulting peptides [13], as well as several studies demonstrated that branched short peptides are more active than lineal one [14–16]. In this study, we investigated how polyvalent presentation of the LfcinB-derived RRWQWR motif affects its antibacterial activity against representative Gram-positive and Gram-negative HCAI bacteria.

RRWQWR motif-derived peptides (linear and branched repetitions) were designed with the aim of developing shorter peptides, to increase antibacterial activity, reduce toxicity, and decrease the coupling reaction steps required and therefore the cost of synthesis. The motif contains positively charged and hydrophobic aromatic amino acids (arginine and tryptophan, respectively), which confer key amphipathic properties at physiological pH.

Cationic and hydrophobic physicochemical properties have been utilized as structural features to improve the function of antibacterial peptides [17]. The crucial physicochemical parameters of the peptides were calculated by using on-line tools. As shown in Table 1, the net charge of the peptides varied from +3 to +12, and this was directly proportional to the antibacterial activity demonstrated. Previous studies have shown that the cationic segments of AMPs facilitate the initial electrostatic attraction and enable interactions with negatively charged components on the bacterial membrane surface [18–20]. However, more recent studies have demonstrated that the relationship between charge and antibacterial activity is nonlinear and that, above a certain threshold (usually +6), increasing the positive charge does not improve antibacterial activity [21, 22]. Even if the results here demonstrate that increasing the number of motif repetitions in the designed peptide induced a higher net charge and that this was related to higher antibacterial activity, it is necessary to consider the relationship between higher antibacterial activity and specificity/selectivity. The hydrophobicity of motif-derived peptides ranged from −3.133 (motif) to −1.207 (LfcinB reference peptide). Therefore, increasing the hydrophobicity and reducing the isoelectric point did not improve antibacterial activity in this study.

Antibacterial activity is related to the nature of bacterial cell membranes, which contain negatively charged lipids in greater abundance than mammalian cell membranes. For this reason, cationic and amphipathic peptides preferentially bind to bacteria by electrostatic attraction, resulting in the specific targeting of bacteria over human cells [23]. Although LfcinB exhibits strong antimicrobial activity, against Gram-positive bacteria [24], Gram-negative bacteria [25], and fungi [26, 27], including multidrug-resistant pathogens, our goal was to establish the effect induced by the polyvalent motif presentation on well-known HCAI pathogens, meaning resistant bacteria. We did not consider cyclic forms, as it has previously been reported that, in spite of the naturally cyclic structure of LfcinB, cyclization is not required for antibacterial activity [9, 28, 29] or* in vitro* cytotoxic effects in some cancer cell lines [9, 30–32]. Our results showed that three of the five molecules studied exhibited antibacterial activity on both microorganisms. The motif and LfcinB reference peptides did not exhibit significant antibacterial activity against these microorganisms, although it was possible to improve their biological activity with linear or branched motif repetitions. Interestingly, the three active molecules (palindromic, dimeric, and tetrameric) exhibited a broad spectrum of activity on both the Gram-positive and Gram-negative species tested, including resistant microorganisms requiring always higher MIC and MBC values for resistance reference strains that for, respectively, sensitive strain (Figure 2; Table 2).

The results demonstrate that palindromic, dimeric, and tetrameric molecules exhibited antibacterial activity, and there was stronger antibacterial activity (defined as molecules with lower MIC and MBC values) with branched motif repetitions for both Gram-positive and Gram-negative microorganisms, as well as for both sensitive and resistant strains (Figures 3 and 4; Table 2).

Interestingly, dimeric and tetrameric molecules have a lower MIC for sensitive* S. aureus* than for sensitive* K. pneumoniae*. Paradoxically, these same molecules on the resistant S*. aureus* strain have a higher MIC than* K. pneumoniae*. These results demonstrate that these cationic molecules (dimeric and tetrameric) exhibit different specificity profiles for Gram-positive and Gram-negative molecules, as well as for sensitive and resistant strains (Table 2). Here we have demonstrated that dimeric and tetrameric molecules exhibited a stronger antibacterial effect on* S. aureus*.

The profile differences may be explained by differences in bacterial membrane components and lipid composition between Gram-positive and Gram-negative species [33] and different responses to environment changes [34, 35] and antibiotic exposure [36–38]. It is well known that different bacterial strains can have unique membrane compositions [38–44].

*S. aureus* is Gram-positive and* K. pneumoniae* is Gram-negative, and both are relevant HCAI pathogens. Gram-positive bacteria have only one membrane (the cytoplasmic membrane that surrounds the cell), while Gram-negative bacteria have two: the cytoplasmic membrane and an outer membrane. This may explain why* K. pneumoniae* required a higher dose of peptide, antibacterial effect similar to that observed with* S. aureus*.

While both Gram-positive and Gram-negative bacteria have a peptidoglycan layer on the outer side of the cytoplasmic membrane, the peptidoglycan layer is much thicker in Gram-positive bacteria. Perhaps this is the reason that the* S. aureus* strains exhibited lower MIC and MBC values (Table 2) due to the fact that thicker peptidoglycan layer makes it easier for peptide to reach the surface membrane and induce formation of pores. SEM microscopy (Figure 5) clearly showed that, after 2 h of treatment with 3x MIC, the* K. pneumoniae* surface membrane exhibited fewer pores and less damage in comparison with* S. aureus* (Figure 4), which displayed dramatic changes under similar conditions. The bacteria surface modifications showed by SEM photography are similar for the changes induced with other antimicrobial cationic peptides on* S. aureus *[45, 46] and* K. pneumoniae *[47] as reference strains. Then, according to the results, our hypothesis is that action mechanism of this branched Lfcin B derived peptides could attach to the bacterial surface and induce the pore formation that disturb the functions of the bacterial membrane as for another cationic peptides was previously described. We are focused on action mechanism studies to elucidate it.

Peptide-lipid interactions are another critical factor. Cationic peptides are facilitated with the negatively charged phospholipids of the microbial membrane. However, it is important to note that the membrane lipid content is diverse. Whereas Gram-positive bacteria contain lipoteichoic acid (LTA) or teichuronic acid (TA), in Gram-negative bacteria it is lipopolysaccharide (LPS) that forms the major lipid component of the outer leaflet of the outer membrane. Consequently, our results could indicate that dimeric and tetrameric molecules have a preference for molecules like LTA and/or TA, expressed on the Gram-positive cytoplasmic membranes, establishing an electrostatic interaction that induces strong antibacterial activity against* S. aureus* strains.

It is possible that branched polyvalent molecules have a higher probability of reaching the bacterial microorganism and establishing electrostatic interactions with anionic molecules and that this depends on the lipid composition of the strain and the Gram classification of the bacteria. Once the dimer and tetramer molecules reach the microorganism, the electrostatic interaction is established, which then leads to microbial lysis and death, perhaps followed by subsequent membrane permeabilization.

The safety of the newly designed molecules is an important aspect to consider for future clinical application. Although the tetrameric and dimeric presentations both demonstrated high antibacterial activity, broad-spectrum activity, and low production cost, their haemolytic activity results indicated that the most innocuous and specific antibacterial molecule was the dimer, at concentrations ≤100 *μ*M, whereas the tetramer has limited therapeutic use at lower concentrations of 12,5 *μ*M. As none of the peptides reached HC_50_, they exhibit (tetrameric 49.3% with 100 *μ*M) and display interesting antimicrobial activity. Our future work will involve detailed study of the mechanisms of antibacterial action used by these dimeric and tetrameric peptides and its specificity profile in terms of security.

## 6. Conclusion

RRWQWR motif repetitions in linear and branched conformations resulted in favorable effects on antibacterial activity against Gram-positive and Gram-negative ATCC strains evaluated in this study. Molecules with branched structures were the most promising even on resistance reference strains.

## Figures and Tables

**Table 1:** Antibacterial peptides derived from LfcinB in this study.

Peptide	Sequence															*m*/*z* [M+H]^+^	Net charge^a^	GRAVY^a^	pI^b^
Motif				^20^ **R**	**R**	**W**	**Q**	**W**	**R** ^25^							986.7	+3	−3.133	12.30
Palindromic					**R**	**W**	**Q**	**W**	**R**	W	Q	W	R			1488.6	+3	−2.678	12.30
LfcinB reference	^17^F	K	C	**R**	**R**	**W**	**Q**	**W**	**R**	M	K	K	L	G	A^31^	1994.7	+6	−1.207	11.74
Dimeric				**(R**	**R**	**W**	**Q**	**W**	**R)**_**2**_	K	Ahx					2198.5	+6	- -	- -
Tetrameric				**(R**	**R**	**W**	**Q**	**W**	**R)**_**4**_	K_2_	Ahx_2_	C_2_				4594.6	+12	- -	- -

^a^Net charge and grand average of hydropathy (GRAVY) were calculated using the Antimicrobial Peptide Calculator and Predictor (http://aps.unmc.edu/AP/prediction/prediction_main.php). ^b^Theoretical pI values were calculated using the Compute pI/Mw Tool (https://web.expasy.org/compute_pi/).

**Table 2:** Antibacterial activity of synthetic peptides derived from the LfcinB RRWQWR motif against HCAI pathogens.

Peptide	*Staphylococcus aureus*				*Klebsiella pneumonia*			
	ATCC 25923 Sensitive		ATCC 33591 Resistant		ATCC 13883 Sensitive		ATCC 700603 Resistant	
	^a^MIC	^b^MBC	MIC	MBC	MIC	MBC	MIC	MBC
Motif	>197 (200)	>197 (200)	>197 (200)	>197 (200)	>197 (200)	>197 (200)	>197 (200)	>197 (200)
Palindromic	74 (50)	148 (100)	9 (6.25)	74 (50)	74 (50)	148 (100)	74 (50)	148 (100)
LfcinB reference	>398 (200)	>398 (200)	100 (50)	199 (100)	>398 (200)	>398 (200)	>398 (200)	>398 (200)
Dimeric	14 (6.25)	110 (50)	55 (25)	110 (50)	27 (12.5)	55 (25)	27 (12.5)	220 (100)
Tetrameric	29 (6.25)	57 (12.5)	57 (12.5)	57 (12.5)	29 (6.25)	115 (25)	57 (12.5)	115 (25)

^a^The minimum inhibitory concentration expressed in *µ*g/mL and (*µ*M). ^b^The minimum bactericidal concentration expressed in *µ*g/mL and (*µ*M). Data are the averages of four independent experiments, each performed in duplicate.
